# Computer vision in surgery: from potential to clinical value

**DOI:** 10.1038/s41746-022-00707-5

**Published:** 2022-10-28

**Authors:** Pietro Mascagni, Deepak Alapatt, Luca Sestini, Maria S. Altieri, Amin Madani, Yusuke Watanabe, Adnan Alseidi, Jay A. Redan, Sergio Alfieri, Guido Costamagna, Ivo Boškoski, Nicolas Padoy, Daniel A. Hashimoto

**Affiliations:** 1 Gemelli Hospital, Catholic University of the Sacred Heart, Rome, Italy; 2 IHU-Strasbourg, Institute of Image-Guided Surgery, Strasbourg, France; 3 Global Surgical Artificial Intelligence Collaborative, Toronto, ON Canada; 4 ICube, University of Strasbourg, CNRS, IHU Strasbourg, France; 5 Department of Electronics, Information and Bioengineering, Politecnico di Milano, Milano, Italy; 6 Department of Surgery, University of Pennsylvania Perelman School of Medicine, Philadelphia, PA USA; 7 Department of Surgery, University Health Network, Toronto, ON Canada; 8 Department of Surgery, University of Hokkaido, Hokkaido, Japan; 9 Department of Surgery, University of California San Francisco, San Francisco, CA USA; 10 Department of Surgery, AdventHealth-Celebration Health, Celebration, FL USA; 11 Fondazione Policlinico Universitario A. Gemelli IRCCS, Rome, Italy

**Keywords:** Translational research, Preclinical research

## Abstract

Hundreds of millions of operations are performed worldwide each year, and the rising uptake in minimally invasive surgery has enabled fiber optic cameras and robots to become both important tools to conduct surgery and sensors from which to capture information about surgery. Computer vision (CV), the application of algorithms to analyze and interpret visual data, has become a critical technology through which to study the intraoperative phase of care with the goals of augmenting surgeons’ decision-making processes, supporting safer surgery, and expanding access to surgical care. While much work has been performed on potential use cases, there are currently no CV tools widely used for diagnostic or therapeutic applications in surgery. Using laparoscopic cholecystectomy as an example, we reviewed current CV techniques that have been applied to minimally invasive surgery and their clinical applications. Finally, we discuss the challenges and obstacles that remain to be overcome for broader implementation and adoption of CV in surgery.

With over 330 million procedures performed annually, surgery represents a critical segment of healthcare systems worldwide^1^. Surgery, however, is not readily accessible to all. The Lancet Commission on Global Surgery estimated that 143 million additional surgical procedures are needed each year to “save lives and prevent disability”^2^. Improvements in perioperative care and the introduction of minimally invasive approaches have made the surgery more effective but also more complex and expensive, with surgery accounting for about one-third of U.S. healthcare costs^3^. Furthermore, a large proportion of preventable medical errors happen in operating rooms (OR)^4^. These observations suggest the need for developing solutions to improve surgical safety and efficiency.

The analysis of videos of surgical procedures and OR activities could offer strategies to improve this critical phase of surgical care. This is especially true for procedures performed with a minimally invasive approach, which is being increasingly adopted globally^5–7^ and heavily relies on the visualization provided by fiber optic cameras. In fact, in minimally invasive surgery the partial loss of haptic feedback is compensated by magnified, high-definition videos acquired by endoscopic cameras^8^. Endoscopic videos guiding surgical procedures represent a direct and readily available source of digital data on the intraoperative phase of surgical care.

In recent years, the analysis of endoscopic videos of minimally invasive surgical procedures has enabled the study of the impact of OR activities on patient outcomes^9^ and the assessment of quality improvement initiatives^10^. In addition, video-based assessment (VBA) is being increasingly investigated for operative performance assessment, formative feedback, and surgical credentialing. However, VBA has mostly remained confined to the research domain given the burden of manually reviewing and consistently assessing surgical videos^11,12^. Expanding on initial successes in minimally invasive surgery, use of video has been growing in open surgery as well^13^.

Computer vision (CV), a computer science discipline that utilizes artificial intelligence (AI) techniques such as deep learning (DL) to process and analyze visual data, could facilitate endoscopic video analysis and allow scaling of applications for the benefit of a wider group of surgeons and patients^14^. Furthermore, while humans tend to grossly assess images qualitatively, computer algorithms have the potential to extract invisible, *quantitative*, and objective information on intraoperative events. Finally, automated, online, endoscopic video analysis could allow us to monitor cases in real-time, predict complications, and intervene to improve care and prevent adverse events.

Recently, several DL-based CV solutions mostly for minimally invasive surgery have been developed by academics as well as industry groups. CV applications range from workflow analysis to automated performance assessment. While analogous digital solutions are being clinically translated and implemented at scale for diagnostic applications in gastrointestinal endoscopy^15^ and radiology^16^, CV in surgery is lagging.

We discuss the current state, potential, and possible paths toward the clinical value of computer vision in surgery. We examined laparoscopic cholecystectomy, currently the most studied surgical procedure for CV methods, to provide a specific example of how CV has been approached in surgery; however, many of these methods have been applied to robotic, endoscopic, and open surgery as well. Finally, we discuss recent efforts to improve access and methods to better model surgical data together with the ethical, legal, and educational considerations fundamental to delivering value to patients, clinicians, and healthcare systems.

## Computer vision for laparoscopic cholecystectomy

Cholecystectomy is the most common abdominal surgical procedure, with almost one million cases performed in the US alone each year^17^. The safety and efficacy of minimally invasive surgery were demonstrated over two decades ago, and laparoscopy has since become the gold standard approach for the removal of the gallbladder. Laparoscopic cholecystectomy (LC) generally follows a standardized operative course, is performed by most general surgeons, and is often one of the first procedures introduced during surgical training. A relatively recent analysis pooling data from more than five thousand patients confirmed the safety of LC, reporting 1.6–5.3% and 0.08–0.14% overall morbidity and mortality rates, respectively^17^. Nonetheless, iatrogenic bile duct injuries (BDIs) still complicate 0.32–1.5% of LCs^17,18^, rates higher than the incidence commonly reported in open surgery^19^. BDIs resulted in a three-fold increase in mortality at one year, a lifelong decrease in quality of life despite expert repair, and were estimated to have an annual cost of about a billion dollars in the U.S. alone^20,21^. Overconfidence in performing this very common surgical procedure and variability in LC operative difficulty have resulted in the scarce implementation of safety guidelines and the consequent non-decreasing incidence of BDI.

Thus, the ubiquity and standardization of LCs have made this procedure an attractive benchmark for CV research and development in minimally invasive surgery^22,23^. In addition, the visual nature and importance of BDI have incentivized both academia and industry to develop CV solutions to solve this well-defined clinical need. Finally, the public release of datasets of annotated LC videos has boosted interest and facilitated research in the field^24^.

### Computer vision analysis

**Fig. 1:**
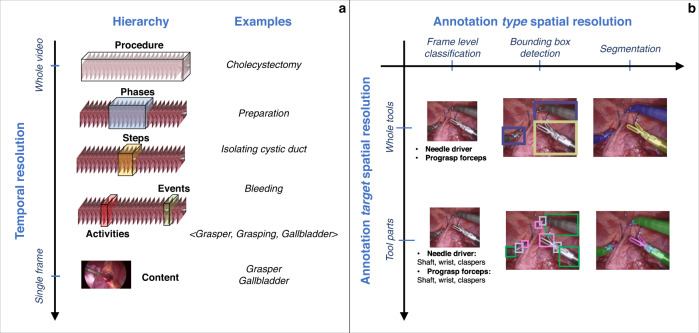
Framework for the analysis of endoscopic videos. Temporal (**a**) and spatial (**b**) annotations at different resolutions are used to model tasks at increasingly finer details.

At the coarsest level, a surgery can be described by identifying the procedure being performed. For example, automatic recognition of the type of laparoscopic procedure from the first 10 minutes of surgical procedures has proven highly effective^25^. Though such applications may not immediately seem clinically relevant, they could serve to several indirect purposes, such as reducing annotation efforts for more specific tasks^26^ or triggering procedure-specific models without human intervention. Once the type of procedure is identified, consensus suggests that surgical procedures can be described both temporally and spatially using a hierarchy of increasingly detailed descriptors or annotations (Fig. 1)^27^. In practice, this hierarchy inherently indicates a natural progression of increasingly complex tasks to annotate and model.

At the coarsest temporal level, an entire surgical video can be classified into phases, broad stages of surgical procedures, which can be further broken down into more specific steps that are performed to achieve meaningful surgical goals such as exposing specific anatomic structures. In 2016, EndoNet first tackled the task of surgical phase recognition using a convolutional neural network (CNN) to automatically extract visual features, including information on the appearance of surgical instruments, from LC video frames^24^. A more detailed temporal analysis could be used to recognize specific activities in surgical videos. Initial works on the topic have formalized surgical actions as triplets comprising the tool serving as the end effector, the verb describing the activity at stake, and the anatomy being targeted (e.g., “grasper, retract, gallbladder”)^28^.

At the briefest temporal extreme, the contents of a single frame, such as the instruments or anatomical characteristics, may be described. When applicable, these contents can be further localized spatially, either loosely with markings such as bounding boxes drawn around structures of interest or precisely with segmentation masks delineating objects with pixel-level accuracy. For spatial annotations, the degree of detail is defined by both the type of annotation (e.g., bounding box vs. segmentation masks) and the target being annotated (e.g., tools or tool parts). Further, the relationships between different localized objects can also be described, for example, to describe the interaction or relative position between instruments and anatomical structures.

Invariably, the limiting factor for most clinical applications is the availability of well-annotated datasets. Coarser labels, such as classifying or qualitatively describing the content of a video sequence rather than segmenting each frame, are less cumbersome to annotate but may appear to serve less directly relevant clinical applications. Nevertheless, coarse-grained labels could be used for: (1) data curation and navigation to streamline the use of video for VBA; (2) education by explaining the contents of a video to trainees; and (3) documentation of and navigation to specific data points to later annotate more details.

### Surgical applications

Fundamental work on CV for temporal and spatial analysis of endoscopic videos allowing automated surgical workflow and scene understanding is being translated to clinically applicable scenarios. LC remains the procedure of choice for demonstrating many such scenarios given its ubiquity and well-defined clinical phenomena; thus, we discuss CV-enabled surgical applications for postoperative video analysis and potential real-time intraoperative assistance in LC. It is important to recognize, however, that such applications are also being investigated for other minimally invasive procedures, gastrointestinal endoscopy, and open surgery^23,29^.

#### Quality improvement

Postoperatively, models for procedure and surgical phase recognition could be used to automatically generate structured and segmented databases to assist with quality improvement initiatives. While such databases would represent an invaluable resource for surgical documentation, research, and education per se, the burden associated with the manual analysis of large quantities of videos presents a considerable bottleneck for adoption. Automated video analysis could be used to digest these large collections of surgical videos, retrieve meaningful video sequences, and extract significant information. For example, full-length surgical videos can be analyzed with phase and tool detection models to identify intraoperative events and effectively produce short videos selectively documenting the division of the cystic duct and the cystic artery, the most critical phase of an LC^30,31^. While this fairly simple approach could be applied to a variety of procedures, adaptation to other use cases would still require considerable development. Very recently, cutting-edge methods have enabled overcoming such barriers by allowing video-to-video retrieval, the task of using a video to search for videos with similar events^32,33^. In addition, models for phase recognition can also be used directly to automatically generate standardized surgical reports of LC. When analyzing such reports based on phase predictions, Berlet et al. found that clusters of incorrectly recognized video frames, i.e. model failures, could indicate complications such as bleeding or problems with gallbladder retrieval^34^. Such events could be linked with the electronic health record to gain insights on patient outcomes after surgery.

#### Operative complexity analysis

CV models can be trained to extract more nuanced information from videos such as surrogates of LC operative difficulty. Since LC operative difficulty correlates with gallbladder inflammation, Loukas et al. trained a CNN to classify the degree of gallbladder wall vascularity yielding performance comparable to expert surgeons^35^. Similarly, Ward et al. trained a CNN to classify gallbladder inflammation according to the Parkland grading scale, a 5-tiered system based on anatomical changes. This classification then contributed to predictions of events such as bile leakage from the gallbladder during surgery and provided insights on how increases in inflammation correlate to prolonged operative times^36^.

#### Operative assessment and feedback

CV models for tool detection have been used to assess the technical skills of surgeons. In this regard, Jin et al. showed that automatically inferred information on tool usage patterns, movement range, and economy correlated with performance assessed by surgeons using validated evaluation metrics^37^. More recently, Lavanchy et al. have proposed to transform automatically extracted tool location information into time-series motion features to use as input of a regression model to predict surgical skills, and distinguish good versus poor technical performance^38^. However, these attempts at automatically assessing technical skills have not been based on existing, validated measures of skill; therefore, more research is required to determine whether automated assessments of skill will supplement or replace traditional assessment methods^39^.

#### Intraoperative decision support

**Fig. 2:**
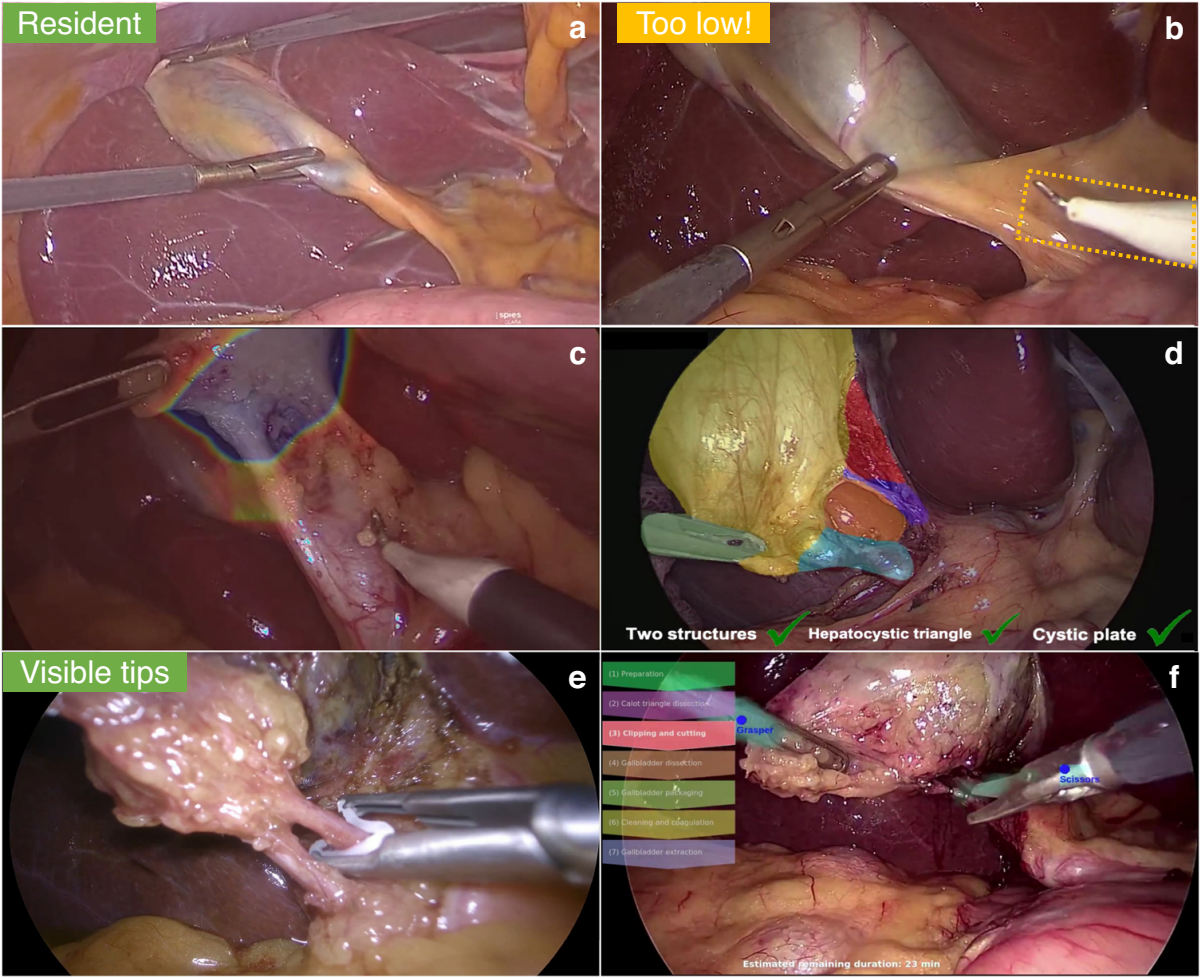
CV-based real-time assistance in laparoscopic cholecystectomy. Overviewed CV models could be used to evaluate the difficulty of a case and whether it is fit for a surgical resident (**a**), to warn surgeons against incising below the appropriate site (**b**), to guide safe dissection (**c**), to automatically evaluate safety measures (**d**), to prevent misapplications of clips (**e**) and to improve OR staff awareness and readiness.

We envision the uptake of AI to assist during minimally invasive procedures (Fig. 2). In this setting, real-time predictions from CV models could be used to guide trainees, enhance surgeon performance, and improve communication in the OR. When starting an LC, CV models could automatically assess the appearance of the gallbladder^35,36^, adjust preoperative estimations of operative difficulty^40^, and suggest whether that case is more appropriate for a trainee or an experienced surgeon. Once the gallbladder is exposed, surgical guidelines suggest using anatomical landmarks to help guide safe zones for incision. For example, Tokuyasu et al. developed a model to automatically detect such key landmarks with bounding boxes^41^.

Similarly, deep learning models could be used to provide a color-coded overlay on the surgical video that could ultimately serve as a navigational assistant for surgeons. Madani et al. have utilized annotations of expert surgeons to train GoNoGoNet to identify safe and unsafe areas of dissection^42^. The endpoint of safe dissection of the hepatocystic triangle is to achieve the critical view of safety (CVS), a universally recommended checkpoint to conclusively identify hepatocystic anatomy and prevent the visual perception illusion causing 97% of major BDIs^43,44^. In this regard, Mascagni et al. have developed a two-stage CV model to first segment surgical tools and fine-grained hepatocystic anatomy to then predict whether each of the three CVS criteria has been achieved^45^.

While automated confirmation of the CVS can provide the surgeon with additional assurance of anatomy, other CV tools can ensure that clips are well placed, and no other structures are inadvertently being clipped. To provide such assistance, Aspart et al. recently proposed ClipAssistNet, a neural network trained to detect the tips of a clip applier during LC^46^. If experienced surgeons may find such assistance unnecessary and even trivial, trainees and early career surgeons may benefit from the reassurance that can be provided by real-time decision-support algorithms such as GoNoGoNet, DeepCVS, and ClipAssistNet. Such algorithms could serve as automated versions of surgical coaches that can facilitate and augment decision-making in the OR^39^.

#### OR team dynamics

At a broader level, real-time workflow analysis could be used to improve communication, situational awareness, and readiness of the whole surgical team. Analyzing surgical videos, phase detection models^23^ and algorithms to estimate remaining surgical times^47^ can help track the progress of the operation to assist OR staff and anesthesia in planning for the current and next case. Furthermore, workflow analysis could help detect deviation from an expected intraoperative course and trigger an automated request for backup or a second opinion. Finally, a visual postoperative summary of the intraoperative events or “surgical fingerprint” could be analyzed with the patient’s preoperative profile to assess the risk of postoperative morbidity or mortality^48^.

## Key enablers for computer vision in surgery

Despite the plethora of methods for automated analysis of LC videos presented in the last few years, few AI-based CV systems have been proposed to analyze other surgical procedures, with most focused on minimally invasive procedures. This hinders clinical impact, to the point that no CV application is currently widely used in surgery.

Reasons for this lack of generalization and clinical translation are manifold but largely center around the availability and quality of data and performance of existing modeling approaches, two key elements for CV in surgery which are intimately intertwined.

### Surgical data

Historically, surgical procedures were demonstrated in front of trainees and peers in operating theaters with stadium-style seating and windows for natural light. Now, however, operating rooms (ORs) are one of the most siloed components of healthcare systems. Information on OR events is usually only reported in surgeon-dictated post-operative notes or indirectly inferred from postoperative surgical outcomes. As such, it has long been difficult to gather actionable insights on intraoperative adverse events (AE), which occur in up to 2% of all surgical cases^49^. Consequently, clinical needs were mostly identified anecdotally by interviewing surgeons and key opinion leaders, a suboptimal practice prone to biases.

#### Variability in surgical data collection

Today, a greater request for surgical documentation, together with the ease of recording endoscopic videos of minimally invasive surgical procedures, have greatly improved our ability to observe intraoperative events and work toward designing solutions to improve surgical safety and efficiency. However, there is still not much uptake around recording and analyzing surgical data. In a survey of members of a large surgical society, Mazer et al. found surgeons recorded fewer than 40% of their cases though wished up to 80% of videos could be captured. Surgeons felt that lack of equipment, institutional policies, and medico-legal concerns were obstacles to recording cases^50^.

Concerns from surgeons and health systems fearing that intraoperative data might be used against them may be unfounded. A recent review on black box recording devices in the OR has suggested that video data predominantly support surgeons in malpractice cases^51^. Thus, institutions have largely begun to implement an individualized approach to video recording that suits their own needs. Some continue to prohibit the storage of video, others allow it for select purposes but with specifically outlined parameters (e.g., scheduled destruction of data every 30 days), while others still encourage video recording and storage for quality improvement, education, and research purposes only. Therefore, institutions should engage in a review of existing policies and engage stakeholders such as risk management officers, malpractice insurance carriers, surgeons, and patients to determine the best local strategy for video recording. Clear institutional rules would guide surgeons who wish to record their cases for any number of reasons, including but not limited to use for surgical data science purposes.

#### Promoting data acquisition through behavioral incentives

Policies and incentives may help to further shift the culture of surgical data collection to favor greater operative data collection and use amongst clinicians who may otherwise not consider the value of intraoperative video and computer vision analyses. Institutions that understand the value of video data can play a role in incentivizing clinicians. As an example, AdventHealth, a large academic health system in the United States (US), partnered with a patient safety organization (PSO) to collect and analyze voluntarily submitted data and provides feedback to clinicians, to improve its quality improvement initiatives around operative feedback^52^. In the US, PSOs were established by the Patient Safety and Quality Improvement Act of 2005 and protect the patient safety work products of voluntarily submitted data for quality improvement purposes from civil, criminal, administrative, and disciplinary proceedings except in narrow and specific circumstances. PSOs are organizations that are independent of a health system and certified by the US Agency for Healthcare Research and Quality (AHRQ).

Furthermore, AdventHealth offered continuing medical education (CME) credits necessary for licensing renewals and ongoing board certification as a further individual incentive to surgeons to record and submit videos and review others’ videos for quality improvement and educational purposes, such as peer review and feedback. By combining statutory reassurance of privacy with individual incentives in the form of CME, this health system has encouraged voluntary submission of video data from a majority of its surgeons. Such protections and incentives should be considered by other health systems to encourage voluntary participation not just in quality improvement programs but also in efforts to develop CV algorithms that can facilitate such quality improvement initiatives. Ultimately, improved incentives and clearly regulatory guidelines could expand the list of publicly available datasets on which CV algorithms could be developed and tested^53^.

#### Limitations in quality of data

It is not merely the quantity of available data that limits the clinical value of computer vision applications but also the quality of that data. While standardized measurements with predictable variability can be utilized in tabular data, such as laboratory values for hemoglobin or creatinine, defining clinical phenomena in surgical videos (i.e., annotation) can be quite difficult. Open surgery presents unique challenges that occur with occlusion of video data from the surgeon’s own movements, necessitating multiple camera angles, additional sensors, or algorithmic approaches to overcome occlusion and consider the added complexity of hand-tool interactions^54–56^.

#### Improving data quality

Clear annotation protocols with extensive annotator training are necessary to ensure that temporal and spatial annotations on surgical videos are clear, reliable, and reproducible. The goals of a given project can help to define the annotation needs and should be clearly established a priori to ensure that appropriate ground truths are established and measured. In addition, annotation protocols should be publicly shared to favor reproducibility and trust by allowing others to collaborate while enabling independent assessment of the ground truth used for training and testing CV models^57^. Ward et al. provide greater detail on the difficulties of annotating surgical video and suggest several key steps that can mitigate against poor or inapplicable model performance related to subpar or inappropriate annotation^58^.

### Artificial intelligence methods

As more and more clinical applications are identified, progressively effective techniques are being introduced to model these applications and bring value to patients. Beyond application-specific modeling, methods are also being developed to help circumvent or mitigate the technical, regulatory, ethical, and clinical constraints endemic to surgery.

#### Methods for better leveraging data

To develop effective clinical solutions, AI models are often trained to replicate expert performance from large quantities of well-annotated data (i.e., fully supervised learning). While leading to unprecedented results in medical image analysis^59^, this learning paradigm is highly dependent on the availability of large annotated datasets. Its sustainability is, therefore, severely limited by issues like strict regulatory constraints on data-sharing and the opportunity cost for clinicians to annotate the data, which make the generation of large datasets far from trivial^60^. These issues are further compounded by the need to well-represent and account for variations between patients (anatomy, demographics, etc.), surgeon interactions (workflow, skills, etc.), and OR hardware (instruments, data acquisition systems, etc.).

Several solutions have been explored to increase the amount of data available, such as using synthetically generated datasets^61^ or artificially augmenting available annotated datasets^62^. Still, sufficiently modeling the range of possible interactions remains an open problem. Recently, approaches for decentralized training (e.g. federated learning) have begun to gain traction^63^, allowing learning from data at remote physical locations, mitigating privacy concerns, and raising the hope of greater data accessibility.

However, even with large quantities of data available, quality annotations are still scarce and expensive to produce. To reduce the dependency on annotations, different solutions have been proposed, leveraging the intrinsic information present in unlabeled data or repurposing knowledge acquired from different tasks and domains. Self-supervised approaches aim at learning useful information from large amounts of unlabeled data by formulating pre-text tasks which do not require external annotations^64^. Semi-supervised approaches also leverage large quantities of unlabeled data but combine them with small amounts of annotated data. This strategy often involves artificial labeling of unlabeled data, guided by some available labeled data^65,66^.

Weakly supervised methods aim to refine readily available but noisy annotations, such as crowd-sourced labels^67^, or to repurpose existing annotations collected for different tasks (e.g. learning surgical tool localization using non-spatial annotations such as binary tool presence^68^). When such annotations are available concurrently with target-task annotations, multi-task training can be carried out (e.g. using tool presence signals to help inform which surgical phase is being carried out and vice-versa)^24^. Alternatively, transfer-learning approaches help repurpose information learned from different tasks and/or domains, for which annotated datasets are more readily available, and apply it to the domain and task of interest (Table 1). A common example is employing transfer learning from large, well-labeled, non-surgical datasets such as ImageNet^69^. Domain adaptation is another popular transfer-learning paradigm when dealing with data coming from *similar* domains as the target one, such as synthetic surgical datasets^61^.

**Table 1:** Common approaches to reduce annotation dependency when learning to perform a task (target task) in a specific domain (target domain).

		Training Annotations		
		Available for target task	Available for different task	Not available
**Training data**	**From target domain**	Augmentation	Weakly-supervised learning Multi-task learning	Self-supervised learning Semi-supervised learning
	**From different domain**	Transfer learning (Domain adaptation)	Transfer learning (e.g. from ImageNet)	

The table reports approaches to facilitate such learning given data from target/different domains, and annotations available for target/different tasks or not available.

#### Methods for trustworthy AI

Even as increasingly effective models are being developed for various clinical applications, technical methods are also required to equip surgical staff with the means to explain AI predictions, interpret the reasons behind them, estimate predictive certainty, and consequently build confidence in the models themselves. These considerations are only now beginning to be addressed in healthcare applications^70^ and are particularly glaring in the case of “black-box” algorithms like deep learning-based methods where the relationships between input and output are not always explicit or well-understood. Here, establishing, formalizing, and communicating causal relationships between features of the input and the model output could help mitigate dangerous model failures and potentially inform model design^71^. It is also important to formalize processes to identify, record, and respond to potential sources of error both before and after model deployment. To this end, Liu et al. present a framework for auditing medical artificial intelligence applications^72^.

Future work could look beyond these issues to methods that can identify when dealing with unfamiliar data (out-of-distribution). Aside from enabling clinicians to make informed decisions based on the reliability of the AI system in specific settings, this could also help researchers recognize and address data selection biases and other confounding factors present in the datasets used to train these models.

#### Methods for AI translation

Each clinical application demands specific conditions to be satisfied in order to be delivered in a timely and appropriate manner in line with existing technical and clinical workflows. As several methods are developed to serve and support various stakeholders during different stages of perioperative care, both hardware and software optimizations will also need to be carefully considered. Acceptable latency, errors, and ergonomic interfaces are all key factors in this discussion. For example, certain optimizations such as running these models with reduced precisions may help dramatically reduce the computational infrastructure needed to deploy these models but may degrade performance. For less time-sensitive applications, cloud computing has been explored for AI-assistance and navigation but is limited by network connectivity^73^.

## Ethical, cultural, and educational considerations

**Fig. 3:**
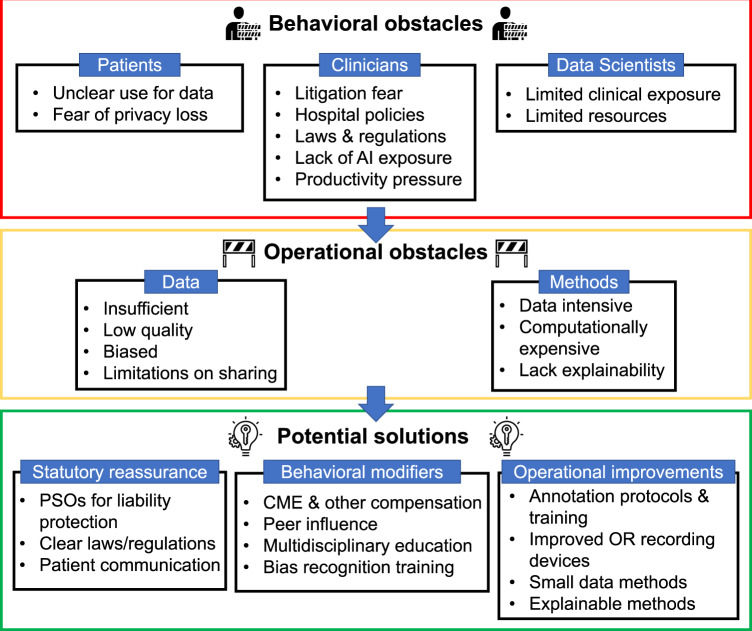
Obstacles and possible solutions for CV in surgery. Behavior and technical/operational obstacles can limit the development and implementation of CV models in surgery. A combination of statutory, behavioral, and operational changes in the regulatory, clinical, and technical environments could result in improvements in the application of CV for surgery. AI artificial intelligence, PSO patient safety organization, CME continuing medical education, OR operating room.

The approaches we have reviewed demonstrate that modern methods have the technical capability to translate computer vision advances to surgical care. However, several obstacles and challenges remain to unlock the potential of computer vision in surgery (Fig. 3). While OR translation, clinical validation, and implementation at the scale of CV solutions are surely fundamental to delivering the promised surgical value, these steps involve multiple stakeholders - from device manufacturers to regulators - and remain largely unexplored today. Here we focus on ethical, cultural and educational considerations important to surgeons and their patients.

Several ethical questions must be addressed, including data safety and transparency, privacy, and fairness and bias^74^. Ongoing discussions are occurring at both the national and international levels to determine how best to protect patients without prohibiting innovations in data analysis that could yield safer surgical care. Considerations for data safety, transparency, and privacy include concepts of informed consent by patients, security of data, and data ownership and access, including whether patients have the right to control and oversee how their personal data is being used.

### Patient perspectives on video data

In a qualitative analysis of 49 patient perspectives of video recording via a hypothetical “black box” system that could capture all surgical data in the OR, 88% of patients felt that any ownership of video data belonged to them as opposed to the hospital at which their care was received or to the surgeon who performed their operations^75^. Regulations around ownership, privacy, and use of identifiable and pseudonymized data vary by country (and even by the state, local, and institutional rules) so research efforts have largely been siloed to individual institutions or local consortia where it may be easier to define who owns data under a given legal infrastructure and how it can be used. As efforts continue to better understand the needs of the field in developing technology that could prove lifesaving for surgical care, it will be critically important to ensure that patients are included and prioritized in discussions that concern the use of data generated through their health encounters.

Patients could be a strong advocate for computer vision research in surgery, as many report perceiving that a benefit of video recording is to enable an objective record of the case to assist in future care and serve as medico-legal protection for both the patient *and* the surgeon. Importantly, patients highlighted their desire for such data to be used for continuous quality improvement^75^. The use of computer vision models such as those we have previously described can facilitate each of these benefits today as context-aware algorithms can automatically index cases for rapid review and post hoc use of guidance algorithms can provide visual feedback to surgeons. Indeed, some institutions are using these technologies to facilitate discussions at weekly morbidity and mortality conferences for quality improvement purposes.

### Bias and transparency of datasets

Additional considerations regarding fairness and bias of datasets that affect model performance and lack of algorithmic transparency have also been highlighted in recent publications^76,77^. Bias in datasets must be acknowledged and considered, especially given that many current and future datasets will be obtained from laparoscopic and robotic platforms that may not be as accessible to low- and middle-income countries. It is also important for researchers to recognize that bias can be introduced at the level of each operation, as surgeons carry with them the influence of their training and prior operative experience in surgical decision-making. The amalgamation of such influences will undoubtedly introduce bias into datasets that could impact model performance and thus the generalizability of CV tools in surgery.

### Collaboration to overcome barriers to computer vision research in surgery

As the importance of bias in datasets and the need for representative, generalizable data has been increasingly recognized, efforts have grown around expanding the collaborative nature of AI research for surgery. For example, the Global Surgical Artificial Intelligence Collaborative (GSAC), a nonprofit organization dedicated to promoting the democratization of surgical care through the intersection of education, innovation, and technology, has been facilitating research collaborations across institutions in the US, Canada, and Europe by providing tools for annotation, data sharing, and model development that meets regulatory standards of each of the participating institutions’ home countries. Focused efforts such as GSAC can lower the barrier of entry for institutions and individuals without significant access to either data or computational resources by facilitating cost sharing, providing infrastructure, and expanding access to both technical and surgical expertise for collaborative work.

### Data science education for clinicians

Finally, education in surgical data science is of paramount importance, both to ensure that current clinicians can understand how computer vision and other AI tools impact their decision-making and patients and to enable future generations to contribute their own insights into developing newer, more sophisticated tools. The Royal College of Physicians and Surgeons of Canada has recently identified digital health literacy as a potential new competency for Canadian physicians in specialty practice, highlighting the importance of new careers that combine medical knowledge with graduate education in AI as well as multidisciplinary clinical teams that incorporate data scientists and AI researchers^78^. A similar conclusion was reached in the UK’s Topol Review on preparing the healthcare workforce for a digital future in the National Health Service (NHS), and the NHS subsequently established Topol Digital Fellowships to teach digital transformation techniques^79^. Institutional, interdisciplinary fellowships are now being established to promote greater clinician literacy in AI topics and greater understanding of clinical problems and workflow by engineers and data scientists. Additionally, institutions such as IHU Strasbourg are offering short, intensive courses in surgical data science to both clinicians and engineers/data scientists to promote interdisciplinary education and collaboration.

## Conclusion

Computer vision offers an unprecedented means to study and improve the intraoperative phase of surgery at scale. As both the clinical and data science communities have begun to converge on advancing research and scientific inquiry on how best to utilize CV in surgery, several proof-of-concept applications of potential clinical value have been demonstrated in minimally invasive surgery. Key efforts to generalize such applications focus around streamlining access to surgical data and better modeling methods, always considering the cultural and ethical aspects intrinsic to patient care. As CV in surgery matures, broader societal involvement will be necessary to ensure the promises of CV in surgery are translated safely and efficaciously to assist in the care of surgical patients.

## Data Availability

Data sharing not applicable to this article as no datasets were generated or analysed during the current study.
